# Effect of Composite Chitosan/Sodium Alginate Gel Coatings on the Quality of Fresh-Cut Purple-Flesh Sweet Potato

**DOI:** 10.3390/gels8110747

**Published:** 2022-11-17

**Authors:** Chit-Swe Chit, Ibukunoluwa Fola Olawuyi, Jong Jin Park, Won Young Lee

**Affiliations:** 1 School of Food Science and Biotechnology, Kyungpook National University, Daegu 41566, Republic of Korea; 2 Coastal Agricultural Research Institute, Kyungpook National University, Daegu 41566, Republic of Korea; 3 Research Institute of Tailored Food Technology, Kyungpook National University, Daegu 41566, Republic of Korea

**Keywords:** purple-flesh sweet-potato, chitosan, sodium alginate, gel coating, preservation

## Abstract

In this study, single-layer coating using chitosan (Ch) and sodium alginate (SA) solutions and their gel coating (ChCSA) formed by layer-by-layer (LbL) electrostatic deposition using calcium chloride (C) as a cross linking agent were prepared to improve storage qualities and shelf-life of fresh-cut purple-flesh sweet potatoes (PFSP). The preservative effects of single-layer coating in comparison with LbL on the quality parameters of fresh-cut PFSP, including color change, weight loss, firmness, microbial analysis, CO_2_ production, pH, solid content, total anthocyanin content (TAC), and total phenolic content (TPC) were evaluated during 16 days of storage at 5 °C. Uncoated samples were applicable as a control. The result established the effectiveness of coating in reducing microbial proliferation (~2 times), color changes (~3 times), and weight loss (~4 times) with negligible firmness losses after the storage period. In addition, TAC and TPC were better retained in the coated samples than in the uncoated samples. In contrast, quality deterioration was observed in the uncoated fresh cuts, which progressed with storage time. Relatively, gel-coating ChCSA showed superior effects in preserving the quality of fresh-cut PFSP and could be suggested as a commercial method for preserving fresh-cut purple-flesh sweet potato and other similar roots.

## 1. Introduction

Purple flesh sweet potato (*Ipomoea batatas*) is a very nutritious root vegetable native to the tropical regions of America. They are an abundant source of carbohydrates, dietary fiber, vitamins including A, B1, B2, C, and E, and minerals including Ca, Mg, K, and Zn [1]. In addition, purple flesh sweet potatoes (PFSP) contain a large amount of anthocyanins, an antioxidant whose long-term dietary intake can prevent cancer, cardiovascular diseases, viral infections, Alzheimer’s disease, and diabetes [2]. The growing consciousness among consumers about what they eat, especially the health benefits, has led to an increase in the consumption of fruits and vegetables. Combined with busy lifestyle patterns, the demand for fresh-cut produce has increased significantly in recent years [3]. Ready-to-use fresh-cut produce is convenient, eliminates consumer waste, and saves time. However, the minimal processing of fresh-cut produce results in tissue softening and discoloration. It increases microbiological deterioration due to the exposed tissues, which makes them vulnerable to metabolism, microbial invasion, and mechanical damage [4]. These factors impact a product’s storage and shelf life [5,6]. Therefore, a suitable packaging technique effective to reduce these factors influence and preserve the quality of fresh-cut produce during marketing and storage is required [4].

Antimicrobial coatings/films (inedible or edible) and modified atmosphere packaging (MAP) have been applied to fresh produce to maintain their qualities and extend their shelf life [7,8,9,10]. In particular, edible coatings have been investigated for their potential to enhance the quality and shelf life of food items [3,11]. Edible coatings can preserve fresh-cut food from mechanical and microbial damage, delay biochemical changes, and enhance their surface appearance [12]. Moreover, edible coatings can meet additional requirements, such as having antimicrobial activity and acting as good moisture and oxygen barriers. These requirements are beneficial for whole or fresh-cut fruits and vegetables that are often prone to microbial harm and highly susceptible to water loss, which causes size shrinkage and texture degradation [5]. Thus, coatings intended for fruit and vegetable preservation are expected to have good gas permeability for typical CO_2_/O_2_ exchange, low water vapor permeability to minimize moisture leakage, and antibacterial properties to inhibit microbial proliferation. It is, however, challenging for a single coating material to satisfy all these requirements [13]

The composite layer-by-layer (LbL) coating technique, which is based on electrostatic deposition technology, was developed to incorporate numerous preservatives derived from various polymer components [5,14]. This approach is based on the alternate deposition of oppositely charged polyelectrolytes in the presence of a cross linking agent, resulting in a novel gel coating with improved properties and functionalities [11]. Due to the effectiveness of the LbL coating technique, its commercial implementation has been suggested for preserving minimally processed fruits. Cationic biopolymers such as chitosan and poly-L-lysine, and anionic biopolymers such as pectin and alginate are commonly used for LbL coating of foods [15]. Alginate is a hydrophilic biopolymer with excellent film-forming properties due to its unusual colloidal properties, including thickening, suspension formation, gel formation, and emulsion stabilization [16]. In addition, sodium alginate coating was beneficial in preserving the post-harvest quality of tomatoes [17] and peaches [18]. However, alginate has no antimicrobial properties, and their poor mechanical properties and water vapor resistance has limited their industrial applications [19]. In contrast, chitosan, a cationic polysaccharide with a high molecular weight and soluble in organic acids, is applicable as a preservative coating material for fruits due to its anti fungal mechanisms [20,21,22,23].

Some studies have examined the effect of alginate and chitosan on fresh-cut melon, mangoes, blueberries [24], guavas, and nectarines [25]. The combination of alginate and chitosan displayed various preservative effects depending on the fresh-cut fruit. However, the application of the sequential coating of chitosan and alginate on fresh-cut purple sweet potatoes has not been studied. This study aimed to investigate the effect of chitosan coating (Ch), sodium alginate gel coating (SA + C), and their composite gel coating (ChCSA) on the quality and shelf life of fresh-cut purple sweet potatoes during refrigerated storage at 5 °C for 16 days.

## 2. Results and Discussion

### 2.1. Effect of Coatings on the Color Change during Storage

**Figure 1:**
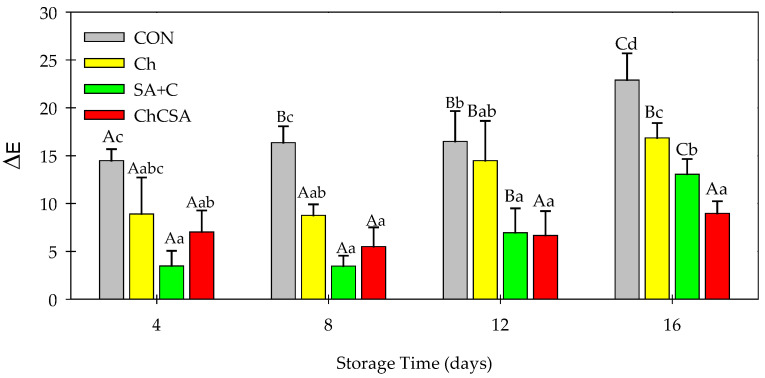
The effect of single-layer and gel coatings on the changes in total color difference value (ΔE) of fresh-cut purple sweet potatoes. Vertical bars represent means and standard deviation. Bars with different alphabets within the same storage day (lower case) or same treatment group at different storage days (upper case) are significantly different (Tukey’s HSD Test, *p ≤* 0.05).

Color is one of the significant visual characteristics of fresh-cut food items. Excessive discoloration often impacts consumer acceptance, and indicates poor performance packaging techniques used to preserve products [26]. The color change (ΔE) value of the samples was used to evaluate discoloration in samples during storage (Figure 1). Change in color was observed in all samples, which was more pronounced in CON (uncoated fresh-cuts). During the first 12 days of storage, no significant difference was observed in coated samples (Ch, SA + C, and ChCSA). However, at the end of storage, notable differences were observed between all samples. The ΔE values for CON, Ch, SA + C, and ChCSA coated fresh-cuts were 22.90, 16.86, 13.05, and 8.97, respectively, indicating that ChCSA gel coating was more efficient in retaining the color of fresh-cut purple sweet potatoes than their single coatings. Biochemical reactions responsible for the degradation of color pigments in sweet potatoes require oxygen and light [1]. The inner and outer film layers of chitosan and alginate, respectively, form a protective barrier on the surface of the coated fresh cuts, which impacts the selective permeability of gas and light [27,28]. Moreover, Ch and SA coatings have been reported to improve the storage quality of various fruits by inhibiting color changes such as browning in papaya, apple, and melon [29,30,31].

### 2.2. Effect of Coatings on Weight Loss during Storage

**Figure 2:**
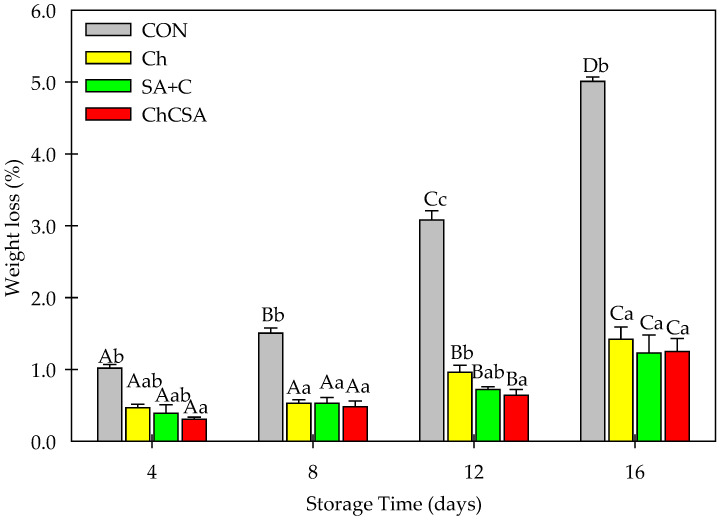
The effect of single-layer and gel coatings on the percentage of weight loss of fresh-cut purple sweet potatoes. Vertical bars represent means and standard deviation. Bars with different alphabets within the same storage day (lower case) or same treatment group at different storage days (upper case) are significantly different (Tukey’s HSD Test, *p ≤* 0.05).

Fresh-cut products are susceptible to weight loss by transpiration [32]. In addition, excessive weight loss reduces valuation and consumers’ perception of purchasing a product [33]. Thus, evaluating weight loss during storage is crucial. Weight loss was gradually increased in all samples according to the storage time (Figure 2). Higher weight loss was recorded in the control samples throughout the storage period, whereas the coated samples had minor weight losses. Significantly, SA + C and ChCSA gel coatings slowed down the weight loss during storage, having the lowest weight loss value (~1.4%) after 16 days of storage. The formation of gel films on the surface of fresh-cut samples improved moisture retention and prevented excess transpiration. Similar to this study, weight loss reduction in coated fresh-cut nectarines [34] and blueberries [24] have been reported.

### 2.3. Effect of Composite Edible Coatings on Firmness

**Figure 3:**
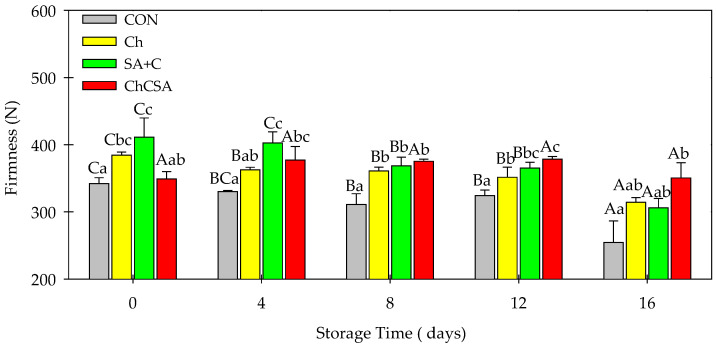
The effect of single-layer and gel coatings on the flesh firmness of fresh-cut purple sweet potatoes. Vertical bars represent means and standard deviation. Bars with different alphabets within the same storage day (lower case) or same treatment group at different storage days (upper case) are significantly different (Tukey’s HSD Test, *p ≤* 0.05).

The firmness of roots and vegetables is also an indicative quality parameter significant for consumer acceptance. The firmness of the control sample decreased throughout the storage period from 341.96 to 254.30 N, whereas CH- and SA + C-coated samples retained their hardness until day 12 (Figure 3). After 16 days of storage, a slight decrease in firmness was observed in Ch (from 384.42 to 314.19 N) and SA + C (411.21 to 306.02 N). However, no noticeable decrease was observed for the ChCSA-coated samples, indicating the beneficial and synergetic effect of multilayer gel coating over their single-layer film coatings. Previous studies reported that layer-by-layer coating enhanced the cell-wall structure and slowed down the cell degradation of fresh-cut products [35,36]. In addition, the combined antimicrobial and adhesion effects of Ch and SA inhibited the production and activities of microbial hydrolytic enzymes associated with cell wall components hydrolysis [11]. Moreover, the use of calcium chloride as a cross linking agent in ChCSA could have further enhanced firmness of coated samples [24].

### 2.4. Effect of Composite Edible Coatings on Microbial Growth

**Figure 4:**
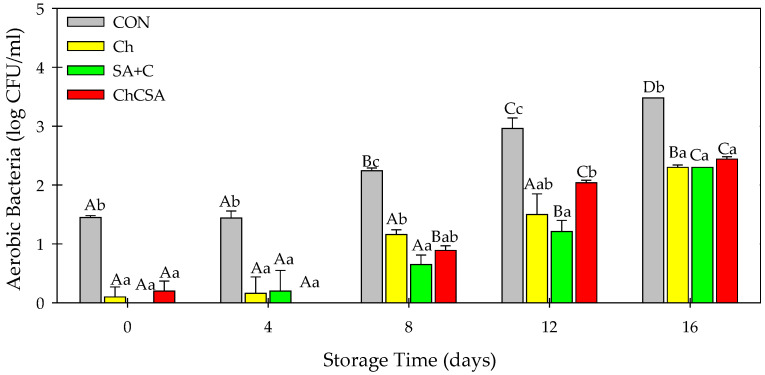
The effect of single-layer and gel coatings on the aerobic bacteria on fresh-cut purple sweet potatoes. Vertical bars represent means and standard deviation. Bars with different alphabets within the same storage day (lower case) or same treatment group at different storage days (upper case) are significantly different (Tukey’s HSD Test, *p ≤* 0.05).

**Figure 5:**
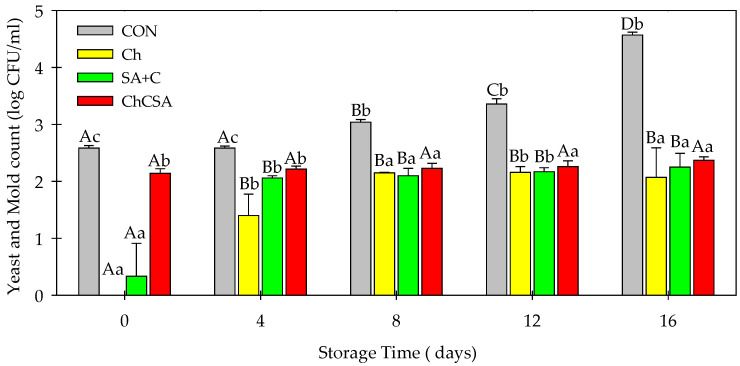
The effect of single-layer and gel coatings on the yeast and mold on fresh-cut purple sweet potatoes. Vertical bars represent means and standard deviation. Bars with different alphabets within the same storage day (lower case) or same treatment group at different storage days (upper case) are significantly different (Tukey’s HSD Test, *p ≤* 0.05).

Microbial contamination is the major reason for the deterioration of fresh-cut products. The presence and growth of microorganisms during product storage and distribution affects food quality and safety [5]. However, some edible coatings have shown barrier properties, inhibiting their proliferation in coated foods [35]. Notably, the application of coatings reduced the initial population of aerobic bacteria (Figure 4) and total fungi (Figure 5). However, an increase in bacteria (3.48 log CFU/mL in CON) and fungi (up to ~4.57 log CFU/mL in CON) were observed in samples at the end of storage. However, all coated samples showed lower microbial concentration. For instance, after 16 days of storage, ChCSA-coated samples had aerobic bacteria and total fungi counts of 2.44 log CFU/mL, and 2.37 log CFU/mL, respectively. The antimicrobial properties of ChCSA coatings could be attributed to the intrinsic bacteriostatic and fungistatic characteristics of chitosan, combined with the oxygen barrier properties of Ch and SA coatings which limited oxygen requirement for microbial proliferation [3,5] 

### 2.5. Effect of Coatings on CO_2_ Production

**Figure 6:**
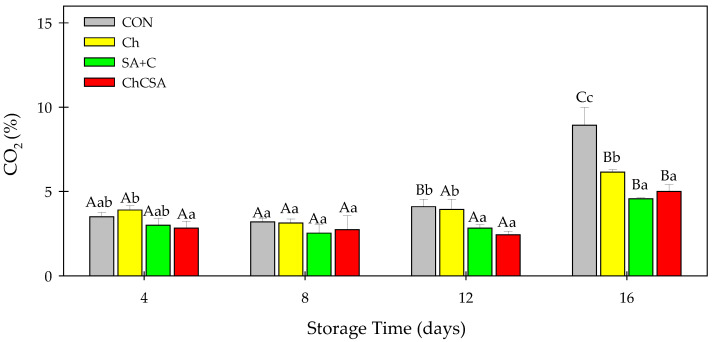
The effect of layer-by-layer and single-layer coatings on the percentage of carbon dioxide gas emission on fresh-cut purple sweet potatoes. Vertical bars represent means and standard deviation. Bars with different alphabets within the same storage day (lower case) or same treatment group at different storage days (upper case) are significantly different (Tukey’s HSD Test, *p ≤* 0.05).

The thin film layer formed by coatings on the food surface controls gas permeability, and provides a delicate balance between inhibiting over-ripening and preventing senescence. In addition, it regulates normal gas exchange to avoid the buildup of CO_2,_ which promotes anaerobic conditions that lead to off flavors [37]. High rate of respiration is one of the problems for fresh-cut products [11]. The composition of CO_2_ in the headspace gas was used to explain the rate of respiration in packaged samples (Figure 6). There were no noticeable differences in CO_2_ production during the first 8 days of storage. Thereafter, CO_2_ concentration slowly increased in all samples until the end of storage. Notably, a sharp increase in CO_2_ production was observed in uncoated fresh-cuts compared to the coated samples. Gel coatings (SA + C and ChCSA) showed better effectiveness in retarding CO_2_ production. High CO_2_ production in fruits corresponds to high oxygen consumption. Thus, the low oxygen permeability of coated samples resulted in tissue respiration, and subsequently, low CO_2_ production [38].

By modifying the gas atmosphere around the fruit tissue, polysaccharide coatings with semipermeable properties on the surface of fruits impede the rate of respiration and ripening during storage, thus retaining the quality attributes of products [39]. Similar gaseous barrier effects of polysaccharide-based coatings on fresh-cut products have been reported [33,40].

### 2.6. Effect of Coatings on Soluble Solid Concentration and pH

**Figure 7:**
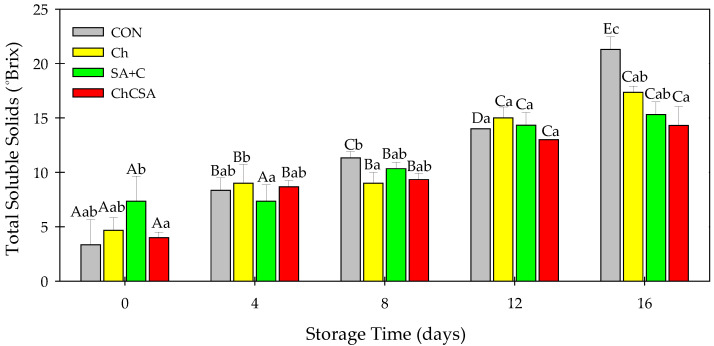
The effect of layer-by-layer and single-layer coatings on the percentage of ^o^Brix of fresh-cut purple sweet potatoes. Vertical bars represent means and standard deviation. Bars with different alphabets within the same storage day (lower case) or same treatment group at different storage days (upper case) are significantly different (Tukey’s HSD Test, *p ≤* 0.05).

As shown in Figure 7, the total soluble solid (TSS), measured as ^o^Brix value, increased in all samples during the storage period. The increase in TSS is due to the conversion of starch and non-starch polysaccharides to simple sugar by hydrolytic processes [27]. After 16 days of storage, TSS was highest in CON (21.3 ^o^Brix) and lowest in ChCSA (14.3 ^o^Brix). Chitosan- and alginate-based coatings were observed to inhibit metabolic and hydrolytic reactions associated with TSS increase in various fruits, including Chinese winter jujube, longan, and fig fruits [27,40,41,42].Thus, low TSS in ChCSA-coated samples could be attributed to the effective combination of Ch and SA, which reduced metabolic reactions and retarded polysaccharides breakdown processes [13].

**Figure 8:**
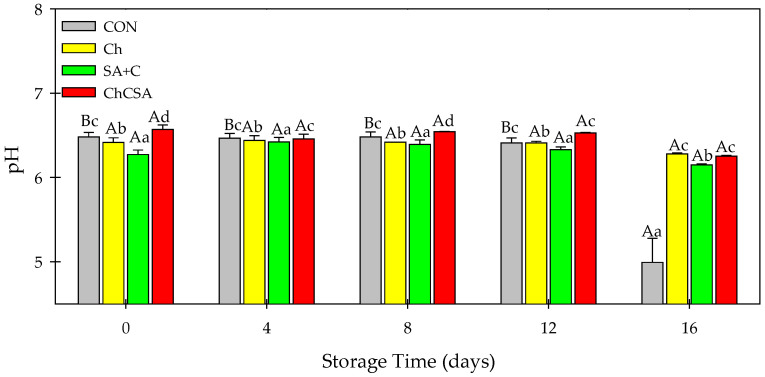
The effect of single-layer and gel coatings on the pH on fresh-cut purple sweet potato potatoes. Vertical bars represent means and standard deviation. Bars with different alphabets within the same storage day (lower case) or same treatment group at different storage days (upper case) are significantly different (Tukey’s HSD Test, *p ≤* 0.05).

Noticeable changes in the pH value of samples occurred after 16 days of storage (Figure 8). CON sample showed a sharp decrease (6.5 to 4.9), while coated samples showed marginal pH changes after the storage period. During post-harvest storage, a decrease in pH is typical and attributed to the production of organic acids by respiratory metabolism [34]. Low pH in CON may be related to the utilization of polysaccharide substrates by microorganisms, which led to the increased production of acidic metabolites [6]. Similar marginal changes in pH value were observed for coated nectarine slices [25] and fresh-cut watermelon [43].

### 2.7. Effect of Coatings on Total Anthocyanin Content and Total Phenolic Content

**Figure 9:**
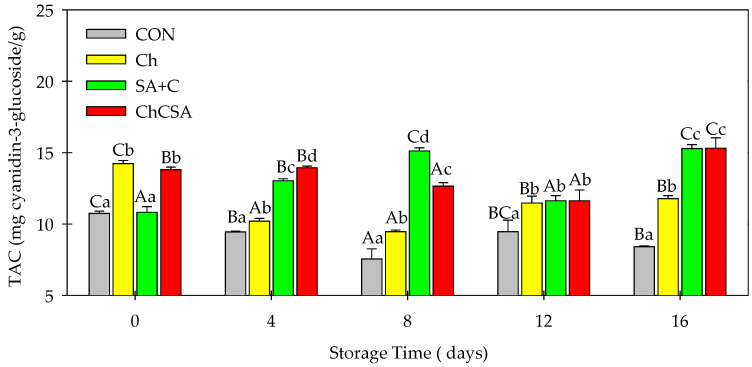
The effect of single-layer and gel coatings on the anthocyanin content on fresh-cut purple sweet potatoes. Vertical bars represent means and standard deviation. Bars with different alphabets within the same storage day (lower case) or same treatment group at different storage days (upper case) are significantly different (Tukey’s HSD Test, *p ≤* 0.05).

Variations in the total anthocyanin content (TAC) were observed in samples during storage, with a more pronounced decrease in CON from 11.1 to 8.4 mg cyanidin-3-glucoside/g after 16 days of storage (Figure 9). These data are consistent with previous studies which showed that anthocyanin content was influenced by the storage time as well as the coating treatment [24]. Moreover, edible coatings have been reported to be beneficial in inhibiting the degradation pathways of anthocyanins in various anthocyanin-rich produce [44,45,46]. Moreover, variations in TAC during storage according to different edible coatings have been previously observed [27,47].

**Figure 10:**
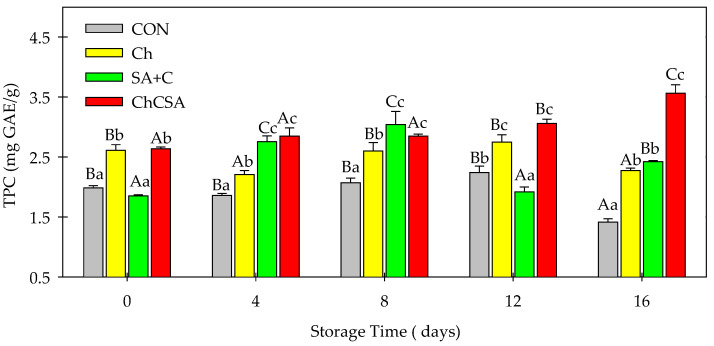
The effect of single-layer and gel coatings on the total phenolic content on fresh-cut purple sweet potatoes. Vertical bars represent means and standard deviation. Bars with different alphabets within the same storage day (lower case) or same treatment group at different storage days (upper case) are significantly different (Tukey’s HSD Test, *p ≤* 0.05).

Similar trends were observed for TPC, in which coated samples prevented phenolic compounds oxidation and degradation, having higher TPC values (2.27–3.56 mg GAE/g) compared to uncoated samples (1.41 mg GAE/g) throughout the storage period (Figure 10). Connor et al. [48] reported that several causes of physiological stress could promote the enzymatic oxidation of phenolic compounds during storage. However, coatings, especially composite coatings, could be beneficial in alleviating these oxidation processes [49]. Similar to the reports of Kou et al. [27], composite ChCSA-coated samples maintained a higher phenolic content throughout 16 days of storage. Moreover, the increase in the phenolic contents could be explained by the effect of Ch/SA coating in promoting phenylalanine ammonia-lyase (PAL) activity which led to the accumulation of phenolic compounds [27].

## 3. Conclusions

This study examined the effect of chitosan-, sodium alginate-, and their composite gel- coatings on the post harvest quality and shelf life of fresh-cut purple sweet potatoes. During 16 days of storage, various physiological and biochemical reactions associated with quality deterioration were effectively controlled in coated samples. For instance, improved quality retention and microbial inhibitions were observed in samples preserved with gel coating formed by Ch and SA multilayer solutions in the presence of CaCl_2_, as a cross linking agent. The observed effects were attributed to enhanced barrier properties and antimicrobial properties, which regulated quality losses by transpiration, respiration, oxidation, and cellular degradation. In summary, ChCSA gel coating achieved the best preservative effect on the post harvest quality and shelf life of fresh-cut purple sweet potatoes, indicating the superiority of layer-by-layer coating over single-layer coating. 

## 4. Materials and Methods

### 4.1. Materials

The experiments were performed with mature purple flesh sweet potato (PFSP) from a farm in Haenam-gun in Korea. The samples were stored at 5 °C. In addition, sodium alginate (32–250 kDa, Duksan Chemicals, Ansan-si, Republic of Korea), high molecular weight chitosan (≥75% deacetylation, Sigma Aldrich, USA), calcium chloride, glacial acetic acid, and Tween-80 were obtained from Sigma Aldrich (St. Louis, MO, USA).

### 4.2. Coating Solutions Preparation

The chitosan solution was prepared according to the method described by [50]. Chitosan powder was mixed with distilled water containing glacial acetic acid (0.5% *v*/*v*) at 70 °C under stirring until fully dissolved to produce a 2% chitosan solution. Finally, the pH of the solution was adjusted to 5.6 with 1 N NaOH.

The sodium alginate solution was prepared according to the method described by [11], with some modifications. Sodium alginate powder was dissolved in 100 mL of distilled water to obtain a 2% concentration. Then, the solution was stirred in a 70 °C water bath for 2 h to dissolve completely. Finally, the sodium alginate solution was cooled at room temperature.

Calcium chloride was used as the cross linking agent to produce gel coatings via layer-by-layer treatment. Calcium chloride was weighed and dissolved in 100 mL of distilled water to obtain a 2% solution. Then, the solution was shaken in the incubator to become dissolved entirely. 

### 4.3. Sample Preparation

Purple flesh sweet potatoes without mechanical injuries or fungal infections were selected and washed in running water. Then, they were peeled and diced to get 1 cm pieces for flesh-cut coating.

### 4.4. Coating Application on the Samples

**Figure 11:**
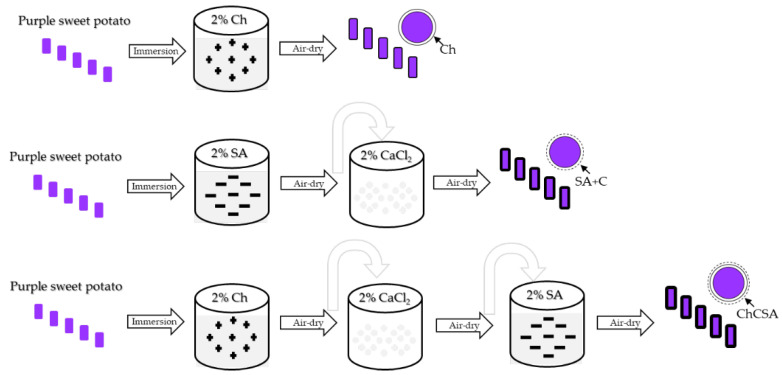
Illustration of coating procedure for fresh-cut purple flesh sweet potatoes.

The coating procedure is illustrated in Figure 11. For a single-layer Ch coating, approximately 2 kg of flesh-cut purple sweet potatoes were dipped in 5 L of Ch solution for 2 min and dried at room temperature for 30 min. For SA gel coating, flesh-cuts were dipped in SA solution, rinsed for 30 s to remove the residual solution, and thereafter immersed in calcium chloride solution and dried. For the multilayer gel coating (ChCSA), the fresh-cuts were dipped in Ch solution for 2 min, rinsed for 30 s to remove the residual solution, immersed in calcium chloride for 2 min, then rinsed for 30 s and finally dipped in SA solution for 2 min before air-drying. Lastly, distilled water was used as an immersion solution for uncoated samples.

For each coating treatment, approximately 200 g of coated fresh-cuts were weighed and stored in triplicate in Ziploc bags (5 °C). Stored samples were removed at 4-days interval during a 16-day storage period and analyzed for quality parameters.

### 4.5. Color Measurement

The surface color of the samples was determined by randomly selecting 3 samples and taking 3 readings for each treatment using a chromameter (CR-300, Minolta Co., Osaka, Japan). The L*, a*, b* value (CIE L a b) system was numerically specified in a three-dimensional spherical space defined by the three perpendicular axes: the L-axis (brightness) ranged from 0 (black) to 100% (white); the a-axis ranged from − a (green) to + a (red); and the b-axis ranged from − b (blue) to + b (yellow). Total color difference (ΔE) was calculated using L, a, and b values with the following equation [51]:

(1) $$\Delta E=\sqrt{{\left(L_{2}-L_{1}{)}^{2}+(a_{2}-a_{1}\right)}^{2}+{\left(b_{2}-b_{1}\right)}^{2}}$$

where subscripts 1 and 2 represent the final and initial readings, respectively at a particular storage interval.

### 4.6. Weight Loss Measurement

The coated and control fresh-cut sweet purple potatoes were individually weighed using a digital laboratory scale (Mettler Toledo, CH/PL 3002) at each data collection interval. The weight loss was calculated as follows:

(2) $$\mathrm{Weight}\;\mathrm{loss}\;(\%)=\left[\frac{w_{\mathrm{in}}-w_{\mathrm{fin}}}{w_{\mathrm{in}}}\right]\times 100$$

where w_in_ and w_fin_ represent the initial and the final weight, respectively, measured at a particular storage interval.

### 4.7. Firmness Measurement

The firmness of the fresh-cut purple sweet potato samples was measured using a texture analyzer (Compac-100, Scientific Co., Tokyo, Japan) equipped with a 3 mm cylinder probe was used to assess the hardness of a fresh-cut purple sweet potato. A puncture test was carried out on a horizontally-positioned sample over a 13 mm hole at the speed of 60 mm/min and a travel distance of 20 mm [3]. The maximum force required to penetrate the sample was recorded for seven randomly selected fresh-cut PFSP per treatment group.

### 4.8. Microbial Analysis

The microbial growth in samples during storage was evaluated by counting the total number of aerobic bacteria and total fungi (yeast and mold). Ten g of the sample was taken aseptically from each treatment and transferred into sterile plastic bags with 30 mL of 0.1% peptone in water. The materials were homogenized in a Stomacher blender (Thomas Scientific, Swedesboro, NJ, USA) and filtered to obtain the sample stock for microbial analysis. Dilutions were done using 0.1% peptone water prior to plating.

Total aerobic bacteria counts were determined by inoculating 100 μL of the diluted extract on the surface of plate count agar (PCA; Becton Dickinson, NJ, USA). The plates were incubated at 37 °C for 24 h. Total fungi counts were determined using the surface inoculation of potato dextrose agar (PDA; Becton Dickinson, NJ, USA), supplemented with ampicillin to control bacterial growth. The plates were also incubated at 30 °C for 48 h [11]. Afterward, the colonies were enumerated, and the results were expressed as the logarithm of colony-forming unit per mL (Log CFU g/mL) of sweet potato.

### 4.9. Carbon Dioxide Production

The samples were analyzed using a digital gas analyzer (Quantek Gas Analyzer Model 902D, Quantek Instruments, Grafton, MA, USA) by inserting the device’s needle probe into the packaging film, enclosing the sample to determine the CO_2_ concentration. The CO_2_ concentration was displayed on the device screen and was computed as the % CO_2_ produced using the following equation [3]:

CO_2_ produced (%) = CO_2fin_ − CO_2in_

where CO_2in_ and CO_2fin_ are CO_2_ concentration on the first day, and at each storage interval.

### 4.10. Soluble Solid Concentration and pH

The soluble solid concentration (TSS) and pH of a sample were measured using the juice extracted from 1 g of the treated sample blended with 20 mL of distilled water in a tissue homogenizer. Soluble solid concentration was determined using a digital refractometer (Atago refractometer model PAL-1, Co., Ltd., Saitama, Japan), and the results were described as ^o^Brix. The pH was measured using a pH meter (METTLER TOLEDO AG8608, Schwerzenbach, Switzerland).

### 4.11. Sample Extraction for Total Anthocyanin Content and Total Phenolic Content

Before analyzing the TAC and TPC of fresh-cut purple sweet potatoes, the sample pieces were frozen (−80 °C) and freeze-dried (FDS8518, Ilsin BioBase Co. Ltd., Dongducheon-si, Republic of Korea) for 7 days. Finally, the freeze-dried samples were ground, and their powder was stored in a freezer at −20 °C using extraction. 0.5 g of dried powder was weighed into a centrifuge tube and dissolved in 10 mL of 50% ethanol. Next, the sample solutions were homogenized for 30 min using an ultrasonic cleaner (JAC-3010; KODO, Hwaseong, Republic of Korea). The tube was placed in a centrifuge (45,000 rpm for 15 min), and finally, clear supernatant was obtained after filtration.

#### 4.11.1. Total Anthocyanin Content

Total anthocyanin content was determined using the pH differential method [52]. Anthocyanin content was measured at the absorbance of 530 and 700 nm at pH 1.0 and 4.5. The results were described as mg of cyanidin-3-glucoside/g (Cy3G/g) of fresh purple sweet potato.

#### 4.11.2. Total Phenolic Content

Total Phenolic Content (TPC) was analyzed using the Folin-Ciocalteu reagent as described in our previous study [9]. TPC values were presented in mg gallic acid equivalents (GAE) per g of fresh-cut purple sweet potato.

### 4.12. Statistical Analysis

The data were analyzed using IBM SPSS (V.20, SPSS Inc., Chicago, IL, USA); The Tukey’s HSP test (honest significant differences) was used to determine the significance of the differences among the treatment means. The results were expressed as the mean ± standard deviation (Turkey’s HSD Test, *p* ≤ 0.05).

## Data Availability

Not applicable.
